# Management of Mobile Aortic Thrombus With Aspirin Monotherapy

**DOI:** 10.7759/cureus.104491

**Published:** 2026-03-01

**Authors:** Neda Salami, Sharmila Raju, Mehnaaz Mohammed, So Un Kim, Harpreet Gill, Sean Hormozian, Aldin Malkoc, Iden Andacheh

**Affiliations:** 1 General Surgery, Arrowhead Regional Medical Center, Colton, USA; 2 Vascular Surgery, Kaiser Permanente Fontana Medical Center, Fontana, USA

**Keywords:** antiplatelet therapy, aspirin monotherapy, mobile aortic thrombus, primary aortic mural thrombus, thoracic aortic thrombus

## Abstract

Mobile aortic thrombus (MAT) is a rare clinical finding that can occur with or without underlying atherosclerotic or aneurysmal disease. Optimal management remains undefined due to limited data. We report the case of a 64-year-old woman with a history of sigmoid diverticulitis and methamphetamine use, incidentally found on computed tomography angiography (CTA) to have a proximal descending aortic mobile thrombus with associated splenic and right renal infarcts. Due to an elevated risk of gastrointestinal bleeding, anticoagulation was not recommended, and the patient was managed conservatively with aspirin 81mg daily and strict blood pressure control. Follow-up CTA at six months demonstrated complete thrombus resolution without recurrent embolic events. This case highlights the potential role of antiplatelet monotherapy in selected patients with MAT when anticoagulation or surgical intervention is contraindicated.

## Introduction

A mobile aortic thrombus (MAT) may arise in association with pre-existing atherosclerosis, aortic aneurysmal disease, drug-induced endothelial injury, or endothelial disorders. In rare cases, thrombus formation occurs without aneurysmal disease, atherosclerosis, or a cardioembolic source, termed primary aortic mural thrombus (PAMT) [1,2]. These lesions are typically diagnosed after symptomatic embolic events involving the cerebral or peripheral arteries.

In a 28-year autopsy series from the University of California, Los Angeles (1955-1983), 95 of 10,671 cases (0.9%) had mural thrombus in the thoracoabdominal aorta. Of these, 48 cases (51%) occurred in aortas of normal caliber and configuration, consistent with PAMT. Despite their rarity, MATs were associated with substantial morbidity, including distal embolization in 17% of cases and fatal major occlusive disease in 6% [3].

In both primary and secondary MAT, no standardized management protocol exists, with options ranging from medical management, endovascular intervention, and surgical intervention. We present a case of MAT, likely related to aortic ulceration or chronic inflammation, successfully treated with aspirin monotherapy, resulting in complete radiographic resolution.

## Case presentation

**Figure 1:**
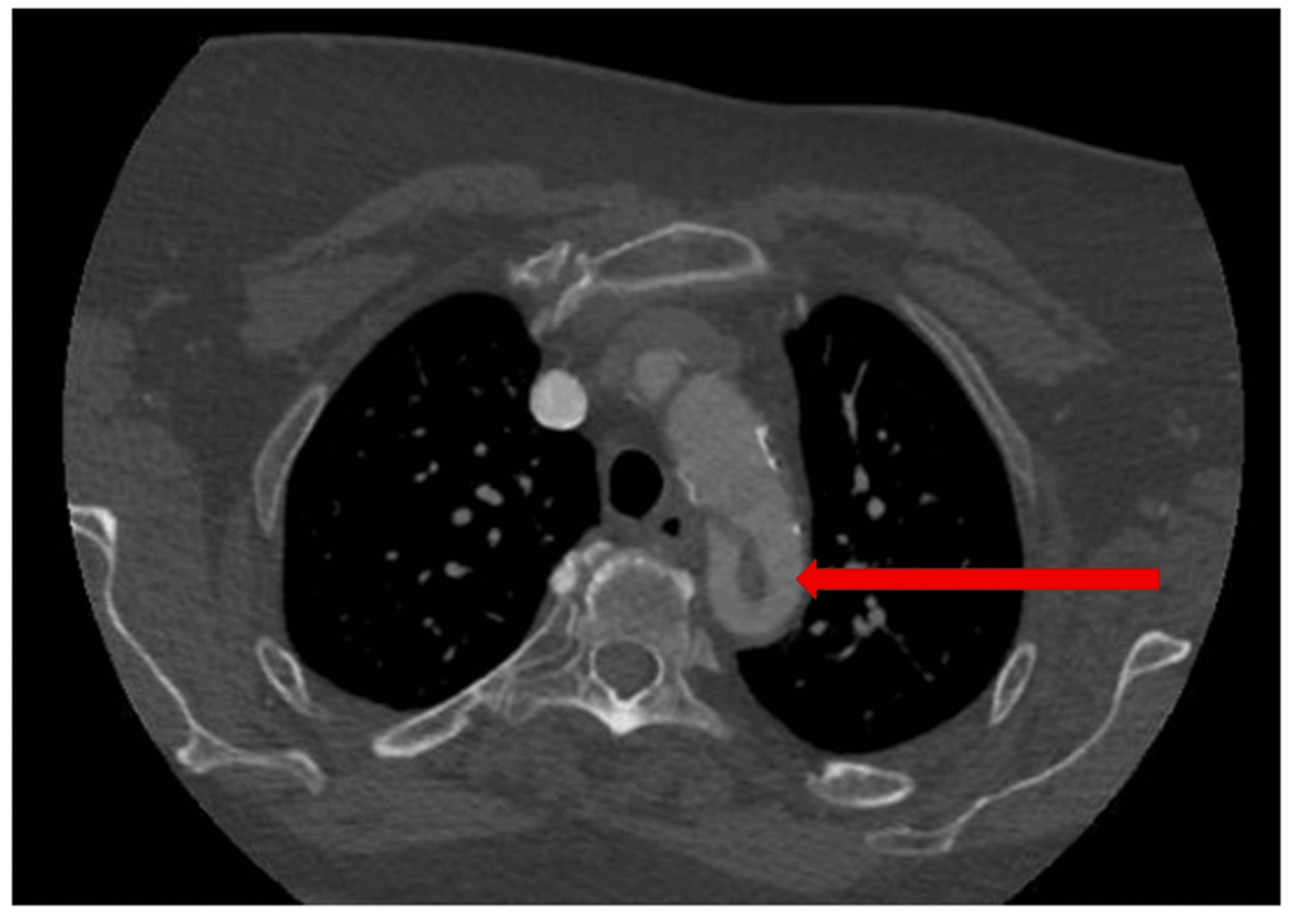
Axial view CTA imaging of the descending thoracic aorta with a mobile thrombus, as indicated by the red arrow, prior to a six-month period of aspirin monotherapy CTA, computed tomography angiography

A 64-year-old woman with a history of sigmoid diverticulitis and methamphetamine use was evaluated after computed tomography angiography (CTA) revealed splenic and right renal infarcts, along with a mobile thrombus in the proximal descending thoracic aorta (Figure 1). She was asymptomatic, without any clinical manifestations of embolic disease on presentation, with a blood pressure of 127/69 mmHg.

Management options discussed included observation with anticoagulation or surgical intervention with thoracic endovascular aortic repair (TEVAR) and left carotid-subclavian bypass. Anticoagulation was not recommended due to a high risk of gastrointestinal bleeding. One month later, she remained asymptomatic except for occasional positional dizziness. She elected medical management, was counseled to avoid blood pressure spikes (including stimulant use), and was started on aspirin 81 mg daily with regular blood pressure monitoring.

**Figure 2:**
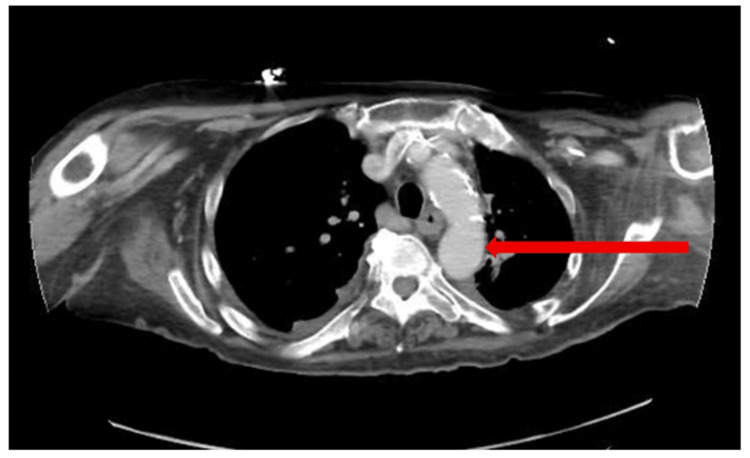
Repeat axial view CTA imaging demonstrating resolution of the descending thoracic aorta mobile thrombus, as demonstrated by the red arrow marking a comparable aortic zone, following completion of six months of aspirin monotherapy CTA, computed tomography angiography

At six months, she remained asymptomatic. Follow-up CTA demonstrated complete resolution of the thrombus (Figure 2) without new vascular lesions or infarcts. She was advised to continue blood pressure control and avoid methamphetamines, cocaine, and smoking.

## Discussion

The work-up of thoracic MAT is often initiated because of embolic signs or symptoms, prompting work-up with imaging studies such as transesophageal echocardiograms (TEE) or CTA studies to be completed. Such imaging modalities can demonstrate embolic complications of aortic thrombi, such as the identification of the splenic and right renal infarcts seen in this case report. Notably, while a TEE may underestimate the size of the thrombus, it highlights thrombus mobility. Additionally, while CTA imaging is less sensitive than TEE, it provides the ability to image the length of the aorta to identify an embolic source [1], as seen in this case report. 

Due to the rarity of mobile thoracic aortic thrombus, a standard treatment protocol does not exist. Therefore, management of patients with such lesions requires risk-benefit discussions with treatment plans tailored to the patient. Multiple treatment strategies have been practiced, such as therapeutic anticoagulation or surgical intervention in patients with persistent thrombus on TEE after two weeks of heparin anticoagulation therapy [1], with surgical intervention options including open versus endovascular treatment [2].

While the use of various anticoagulants has been suggested in the medical management of MAT, including unfractionated heparin, low molecular weight heparin, and warfarin, with durations of treatment individualized to patients based on hypercoagulability risk factors [2], the sole use of antiplatelet therapy with aspirin has not been previously studied. In patients who are appropriate surgical candidates and are deemed to benefit from surgical intervention, TEVAR has demonstrated promising outcomes for the prevention of embolic events in multiple case studies, despite potential risks of intervention. For example, in an eight-year prospective study conducted by Bavaria et al., looking at the risk of ischemic stroke in patients undergoing TEVAR procedures, the benefits of reducing the risk of thromboembolic events secondary to MAT outweighed the potential risk of ischemic strokes with procedural intervention [3,4]. The multitude of proposed treatment alternatives underscores the importance of considering patient risk factors when formulating a treatment plan. In the case described, surgical options were discussed with the patient, but she opted for medical management. Moreover, given her acute bout of diverticulitis, the general surgery team was reticent to initiate warfarin or oral thrombin inhibitor therapy due to the potential for gastrointestinal bleeding. With aspirin monotherapy alone, this patient saw resolution of the MAT.

## Conclusions

This case highlights the successful management of a mobile thoracic aortic thrombus with aspirin monotherapy in a patient with a history of substance use and recurrent diverticulitis, in whom therapeutic anticoagulation was not recommended. Given the risks associated with anticoagulation and surgical intervention, careful patient selection and close follow-up are essential for guiding treatment decisions. Of note, while a hypercoagulable work-up was not assessed in this case study, such clinical studies may be indicated in the management of aortic thrombus disease to further identify individual patient risk factors, particularly for the prevention of recurrent disease. The complete resolution of the thrombus on follow-up imaging emphasizes the potential role of medical management with antiplatelet therapy in select cases. Further studies are warranted to better define optimal treatment strategies for similar presentations.
